# Supplementary figures and images for: Shape analysis of gamma rhythm supports a superlinear inhibitory regime in an inhibition-stabilized network
Source: PLoS Comput Biol. 2022 Feb 14;18(2):e1009886. doi: 10.1371/journal.pcbi.1009886 (PMC8880865; doi:10.1371/journal.pcbi.1009886)

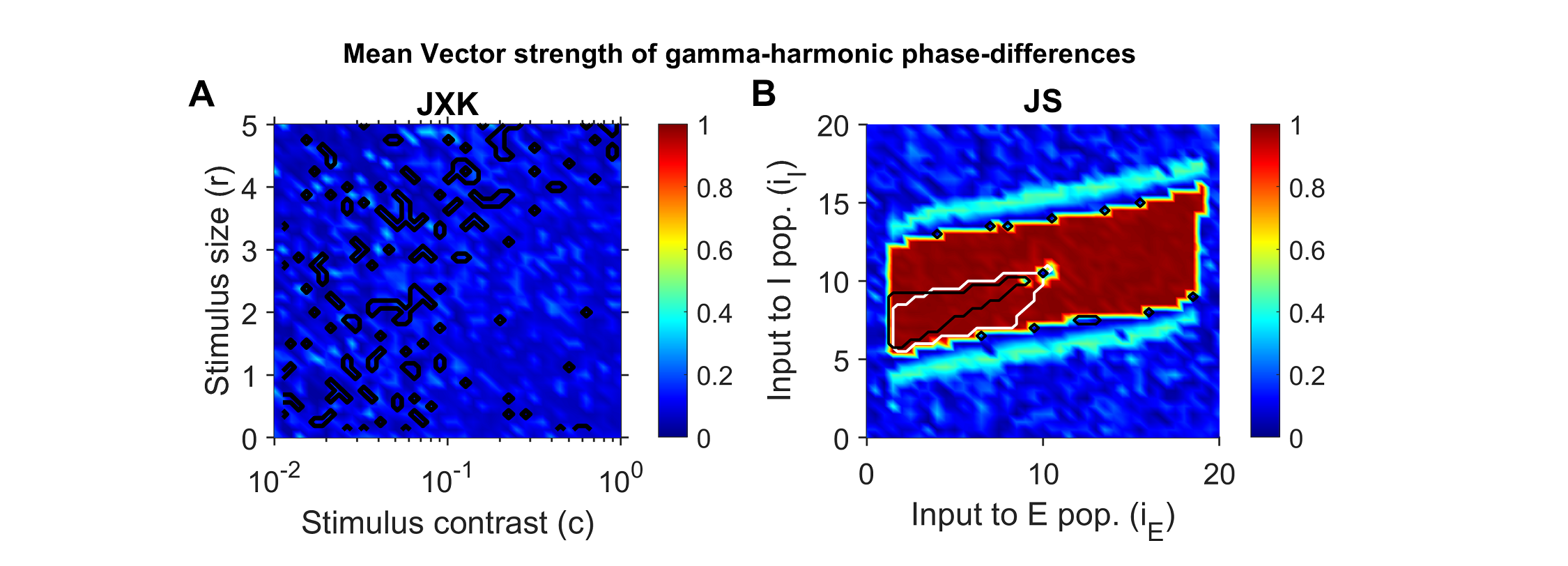

Supplement: S1 Fig — (A) Time-averaged direction vector strengths, averaged across 50 iterations of JXK model simulation for the -rE-rI proxy, computed similarly as for the LFP data. The low vector strengths suggest that the near 180° phase differences identified ‘in-regime’ points were likely not produced by consistent arch-shaped waveforms in subsequent cycles but arose from much rarer sharp troughs in the proxy traces. (B) The mean vector strength computed from the simulation of the JS model for the -rE-rI proxy. For input combinations that generate oscillations by self-oscillation, subsequent gamma cycles have the same shape, resulting in vector strength of 1. The white contour surrounds the superlinear regime of the JS model as in Fig 7F. The black contours in (A) and (B) encircle the input combinations that satisfied our ‘in-regime’ criterion on phase differences as in Figs 6F and 7F. (TIF) [file pcbi.1009886.s001.tif]

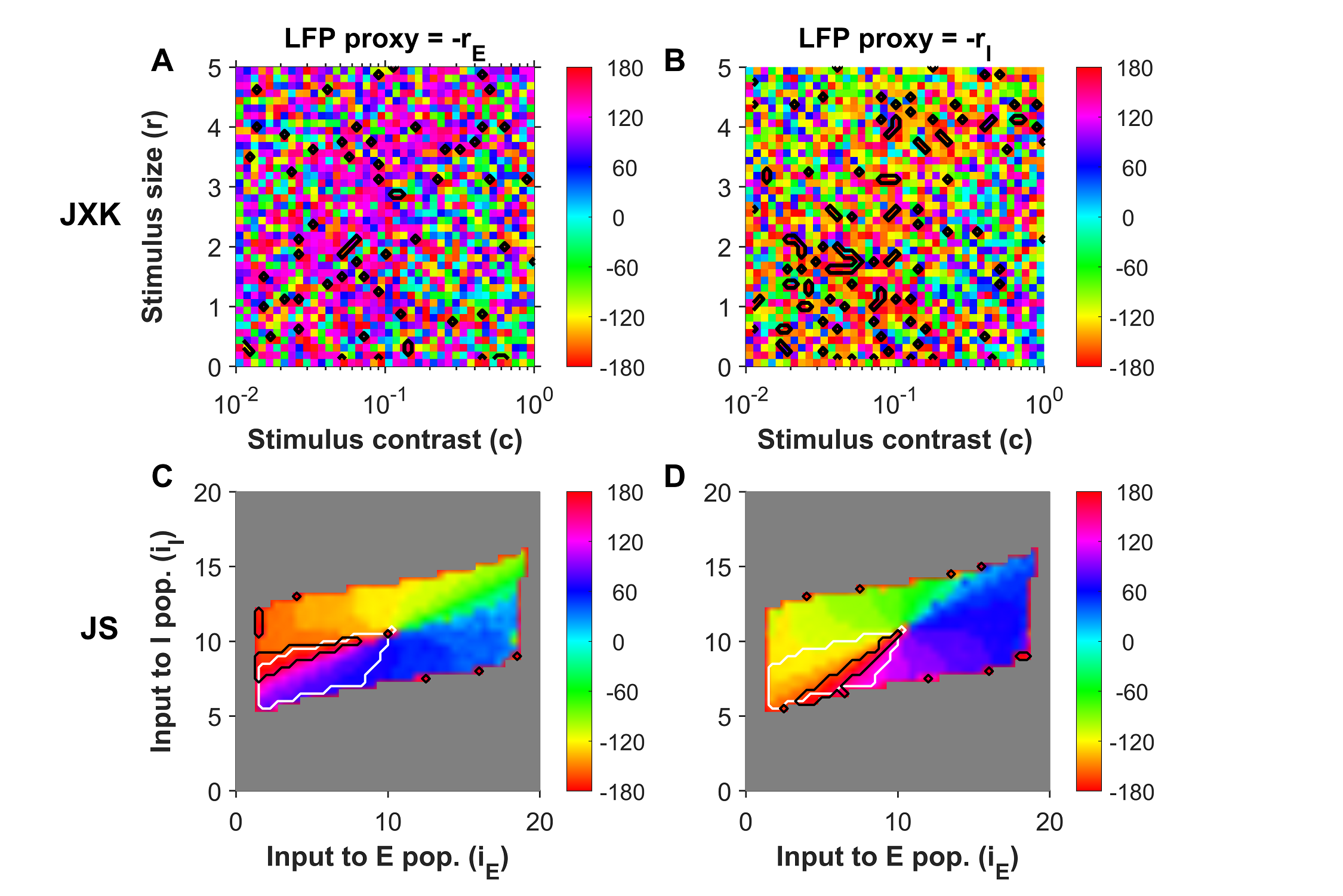

Supplement: S2 Fig — (A) Phase difference of gamma and its harmonic in the JXK model computed for each stimulus condition (as in Fig 6F) but using only E population activity to compute the LFP proxy. (B) Gamma-harmonic phase differences in JXK using I population activity alone. (C) Phase differences in JS model using E population activity only. (D) Phase differences in JS using I population activity only. Black contours encircle stimulus conditions identified as ‘in-regime’. White contours in (C) and (D) mark the superlinear regime of the JS model. (TIF) [file pcbi.1009886.s002.tif]

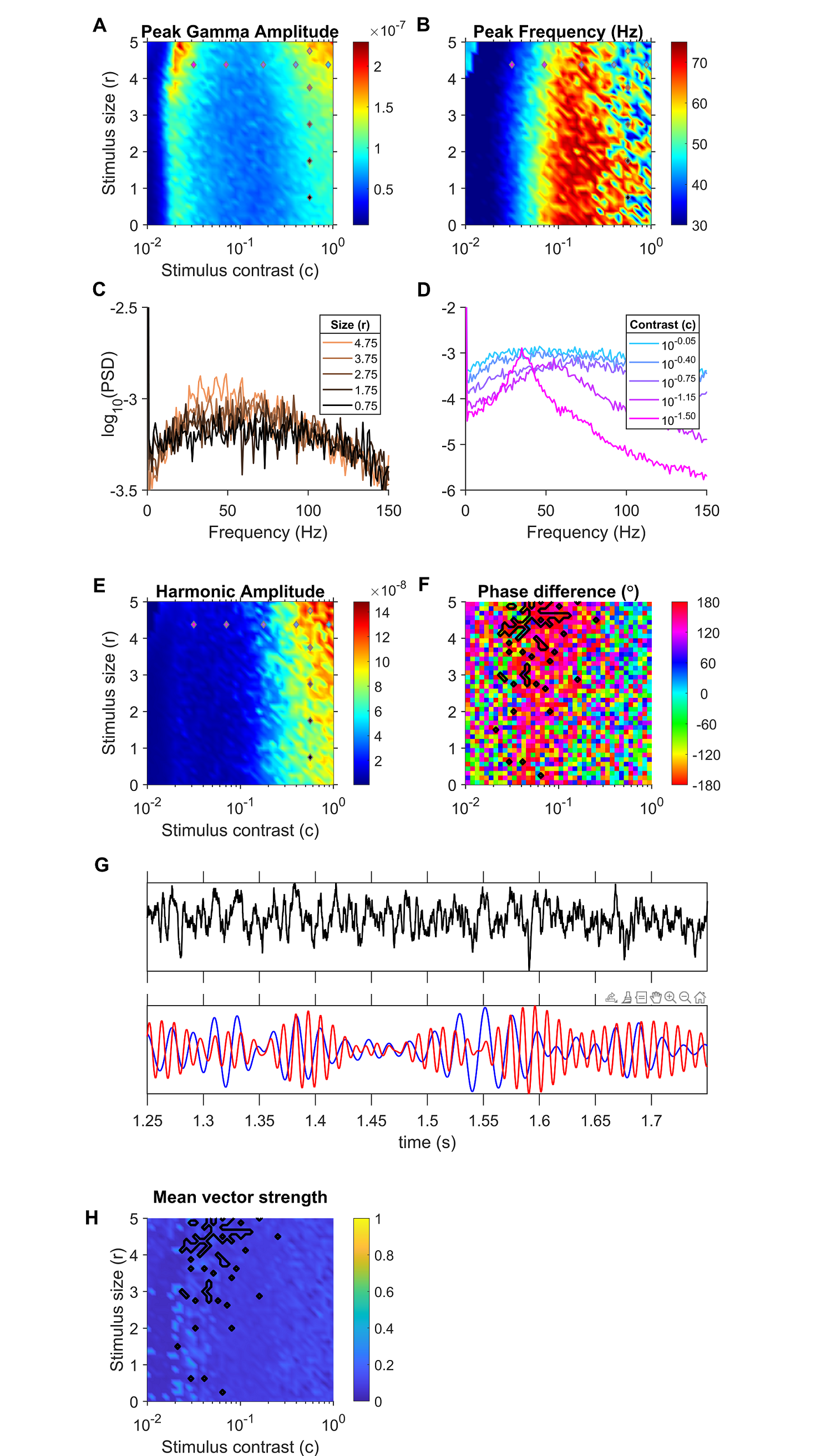

Supplement: S3 Fig — JXK model was simulated for an example input combination with a modified activation function obtained by replacing ⌊rI⌋ with ⌊rI⌋nn with n = 2.5 (A-G) Same as Fig 6A–6G (H) Same as S1A Fig. (TIF) [file pcbi.1009886.s003.tif]
