# Supplementary material for: Shape analysis of gamma rhythm supports a superlinear inhibitory regime in an inhibition-stabilized network
Source: PLoS Comput Biol. 2022 Feb 14;18(2):e1009886. doi: 10.1371/journal.pcbi.1009886 (PMC8880865; doi:10.1371/journal.pcbi.1009886)
Supplement: Attachment — Submitted filename: Response to Reviewers.docx [file pcbi.1009886.s004.docx]

# ***Reviewer comments and Authors’ responses (inline):***

# *Reviewer #1:*

>> We would like to thank the reviewer for providing valuable suggestions and pointing to corrections. Our responses to their comments are presented below.

## *Major comments:*

*— Figures 2 and 5 do not convey the message that wants to be send (or it is difficult to understand). With the aim of improving interpretability, both figures should be rethought to represent the main message better. If, for any reason, the authors want to keep the plots as they are, I suggest to add additional panels with the distribution of values showed in the Y axis of both figures.*

>> We appreciate this suggestion. We have now plotted histograms of the peak frequency ratios and phase differences in Figures 2 and 5 respectively to illustrate the clustering of these quantities about 2 and 180 degrees across stimulus conditions. We have updated the description of these Figures in the Results section.

*— The code in the GitHub repository does not work directly after downloading the folder and follow the steps specified in the Readme. A missing function called mtspectrum is raising an error. If it is part of a mathwork toolbox or has been downloaded from another repository, this should be specified in the Readme, guaranteeing that everyone can reproduce the figures.*

>> Thank you for pointing this out. “mtspectrum” is a function available in a toolbox called Chronux. We have updated the documentation of the repository with the list of the required toolboxes.

## *Minor comments:*

## *— In line 649 the equation needs to be corrected, the words are badly formatted.*

>> The equations have now been reformatted and the statements corrected.

*— In line 598: the references to the figures needs to be corrected, there exists a reference to a missing panel.*

>> The figure references have been corrected. Thank you for pointing this out.

*— I suggest to invert the order of the model parameters in Table 1, such that they match the order they appear in the text and figures, improving consistency and readability.*

>> Thanks for the suggestion. The model parameters are now listed JXK first followed by JS as they occur in the text.

*— Figures 6 and 7 correspond to the same analysis with different models. Consistency between both figures would improve interpretability: please check title style in panels C and D, and box style in panel G.*

>> Thanks for pointing this out. Figure 6 has been reformatted to be consistent with Figure 7.

# *Reviewer #2:*

*This work prevents a well thought out and relevant comparison of competing models of stimulus-induced gamma activity in comparison to a previously published dataset of gamma activity in macaque. The distinctive oscillation shape, linked to a fixed phase relationship between the gamma and harmonic, is shown to be reproduced sufficiently well in only one of the two models tested. While the results are already suitable for publication with minor changes, a bit of additional expansion of the analysis and simulations could significantly enhance the impact of this work, completing the picture of the experiment-model match (or lack thereof).*

>> We thank the reviewer for the succinct description of our study and appreciate the apt suggestions. Our responses to the reviewer’s comments and a description of some changes are presented below.

*Major comments:*

1. *In both model and experiment analyses, the authors essentially discard all information about the variability of phase relationships (with the exception of the Rayleigh test to check for nonuniform phase). The vector strength of the circular average of phases could provide a valuable additional comparison (averaged either within or across trials or both). This might also aid in the discussion of whether the stochastic driven model comes close to reproducing the observations – the current explanation that identifies and then dismisses isolated ‘in-regime’ areas in the parameter space feels a bit artificial, although the explanation of the insufficiency of this model makes sense.*

>> This was a great suggestion. Thank you very much. We have added mean of trial-averaged vector strengths for different stimuli in the LFP data in Figure 5, and the same for the JXK and the JS models. We also mention this in the last paragraph under the section *Gamma oscillations in the JXK model* under Discussion.

2. *The authors mention they have some results on models with differently shaped activation functions, but do not show those here. Although fully exploring that space of models would certainly be a full new result in itself, a brief presentation of a variation or two would be very enlightening here, in particular to disentangle the two contrasts of linear/superlinear and persistent/decaying oscillations. A few traces in a supplemental figure, referenced in the relevant discussion in the main text, would be ideal here.*

>> Thank you for the suggestion. We had indeed tried a variety of activation functions, but had found that the model behavior changed a lot depending on the choice of activation function. Based on this, we speculated that there could be some regimes where these models also produce the desired gamma shape. However, for the functions we tried, we failed to find an appropriate one. We have included a supplementary figure S3 showing our results for the JXK model-variant with a power law non-linearity applied to lateral inhibition. The results were similar for all the non-linearities we tested, which included power laws and sigmoids. Yet, we cannot rule out the possibility of non-linear forced oscillating system generating consistent arch-shaped gamma without a systematic analysis as rightly pointed out in your comment.

We have mentioned this in the Discussion section under *Gamma oscillations in the JXK model*.

## *Minor comments:*

*L48-49: is there a difference between center-frequency and peak-?*

>> We had used the terms interchangeably. Now we use ‘peak frequency’ throughout the paper for consistency.

*L65: “phase does not vary linearly” – with respect to what?*

>> We meant linearly with time. We have made the correction now.

*L158: the trial averaging (over the stimulus interval) should be briefly explained here to more clearly distinguish it from the averaging across electrodes*

>> Thanks for pointing this out. We agree and have now described the time-averaging and electrode-averaging steps briefly in the Results text to alleviate potential confusion.

*Figure 4: If possible, the phase axis should only span a 180 degree interval to more clearly show the possible domain of phase differences*

>> We did not fully understand this suggestion. As shown in row 4 of Figure 4, a gamma-harmonic phase difference of 270 degrees produces a distinctly different waveform. Hence, it is necessary to span the entire range of phase differences from 0 to 360 degrees, and so, we have not changed the phase axis range.

*L197: a brief summary of the structure of the two models would be helpful in the main text here – what are the populations represented and how is the stimulus input represented in the model?*

>> Thank you for the suggestion. We have now added more description. However, since a detailed description would have required much some elaboration, we have specified a reference pointing to the section *Studying non-sinusoidal waveforms in mean-field models of gamma* in Materials and Methods where a detailed description the model structure and the representations of stimulus contrast and size features are provided.

*L244: “inhibitory inputs are superlinear” – more accurate to say the inhibitory population response function is in a superlinear regime?*

>> The statement has been corrected as suggested.

*Figure 7A-B: markers are very hard to distinguish, maybe zoom into the parameter space*

>> Thank you for pointing this out. The marker and trace colors have now been changed for better distinguishability. We have also added a zoomed-in of the in-regime range showing the markers, as suggested. Furthermore, we have updated the captions for Figures 6 and 7 to include a description of the choice of example points to alleviate confusion.

*Figure 8: While this test of alternative LFP proxies is important, I think it could be moved to a supplement to keep the focus on the main results. The repeat of the lack-of-fit of the JXK model here especially adds very little.*

>> Thank you for this suggestion. We have moved this Figure to Supplementary materials.

*L311: Is it possible to interpret the effects of conductances on gamma shape in spiking models as operating through the same shifts in input/response regimes shown in this work?*

>> We believe it to be possible to describe the effects of conductances on gamma shape in a spiking model using the input regimes identified on an equivalent rate model. The rate models considered in our study condense the synaptic and membrane excitability dynamics of different populations into a much-reduced form. Hence, the synaptic dynamics are not explicitly accessible for manipulation. However, any perturbations in magnitude of synaptic conductances of lateral connections in spiking models are equivalent to changes to the corresponding connectivity weights ’W_XY_’ and, in that sense, we could talk about the conductances operating through similar shifts in input/response regimes as shown here. We have added a line regarding this in the paper.

*L389-396: this summary of why the JXK ‘in-regime’ responses are not good fits should move to results, regardless of whether it can be expanded quantitatively using the vector strength analysis mentioned above.*

>> Thank you for this suggestion. We now describe, in the section ‘*Gamma waveform in JXK model*’ under Results, the occurrence of sporadic sharp troughs as the explanation for potentially spurious ‘in-regime’ phase-differences in some input combinations despite the variedly distorted waveforms seen in the bursts. We have added the results of the vector strength analysis from Supplementary Figure S1, as suggested.

*Additional discussion: It would be useful to briefly mention and compare the present superlinear model regime to the supralinear stabilized network regime of (Ahmadian, Rubin and Miller, 2013) – I’m not sure they’re directly comparable, but it seems there may be a contrast where both E and I are superlinear in the SSN vs I only in the JS model?*

>> We have now added a discussion in the section *Gamma oscillations in the JS model* under Discussion.

*L659: equation formatting*

>> Thank you for pointing this out. All equations have now been uniformly reformatted.
